# Feasibility of Using H_3_PO_4_/H_2_O_2_ in the Synthesis of Antimicrobial TiO_2_ Nanoporous Surfaces

**DOI:** 10.1155/2021/6209094

**Published:** 2021-12-11

**Authors:** Benjamín Valdez-Salas, Ernesto Beltrán-Partida

**Affiliations:** Departamento de Corrosión y Materiales Avanzados, Instituto de Ingeniería, Universidad Autónoma de Baja California, Blvd. Benito Juárez y Calle de la Nomal S/N, Mexicali B.C. 21040, Mexico

## Abstract

Ti6Al4V alloys are the primary materials used for clinical bone regeneration and restoration; however, they are substantially susceptible to biomaterial-related infections. Therefore, in the present work, we applied a controllable and stable oxidative nanopatterning strategy by applying H_3_PO_4_, a weaker dissociating acid, as a substitute for H_2_SO_4_ in the classical piranha reaction. The results suggest that our method acted as a concomitant platform to develop reproducible diameter-controlled TiO_2_ nanopores (NPs). Interestingly, our procedure illustrated stable temperature reactions without exothermic responses since the addition of mixture preparation to the nanopatterning reactions. The reactions were carried out for 30 min (NP14), 1 h (NP7), and 2 h (NP36), suggesting the formation of a thin nanopore layer as observed by Raman spectroscopy. Moreover, the antimicrobial activity revealed that NP7 could disrupt active microbial colonization for 2 h and 6 h. The phenotype configuration strikingly showed that NP7 does not alter the cell morphology, thus proposing a disruptive adhesion pathway instead of cellular lysis. Furthermore, preliminary assays suggested an early promoted osteoblasts viability in comparison to the control material. Our work opens a new path for the rationale design of nanobiomaterials with “intelligent surfaces” capable of decreasing microbial adhesion, increasing osteoblast viability, and being scalable for industrial transfer.

## 1. Introduction

Titanium (Ti) and its alloy (Ti6Al4V) are the main biocompatible metallic options currently used to promote bone formation and restoration [1]. However, contamination by microbial adhesion can negatively compromise Ti effectiveness and clinical success. Promising strategies have been reported to generate nanopatterned surfaces to control microbial adhesion. For example, roughness in texture, deposition of antimicrobial nanocoatings, and nanoscale tuned surfaces [2, 3]. Of particular interest, the fabrication of controlled-sized TiO_2_ NPs has emerged as a current trend for controlling microbial adhesion and colonization [4]. Interestingly, chemical oxidative nanopatterning has proven to be a versatile strategy for the development of controlled NP tuned surfaces [5]. Thus far, the H_2_SO_4_/H_2_O_2_ system (piranha solution) is the optimal etching/oxidative protocol for the generation of reproducible NPs and the activation of metallic surfaces. Nonetheless, the mixture with strong acids exacerbates an extreme exothermic reaction that acts violently after organic matter contact (at low concentrations) and on metallic surfaces [6]. Consequently, it is substantial to develop a stable and nonexothermic etching solution for chemical nanopatterning capable of producing NPs on Ti6Al4V surfaces. Thus, by applying H_3_PO_4_, a weaker dissociation acid of pKa lower than H_2_SO_4_ [7, 8], we could reduce the exothermic reactivity on Ti6Al4V without disturbing the formation of reproducible NPs.

Therefore, our work aims to synthesize diameter-controlled and reproducible NPs on Ti6Al4V to reduce microbial adhesion and promote early osteoblast growth using an oxidative nanopatterning procedure with H_3_PO_4_/H_2_O_2_. This strategy is an excellent alternative to the piranha solution since it is more stable, ecofriendly, and nonexothermic suspension without requiring heating conditions.

## 2. Materials and Methods

### 2.1. Synthesis and Characterization of NPs

Ti6Al4V foils (ASTM F136) with 10.0 mm^2^ and 1 mm thickness were polished with SiC emery paper following 1-*µ*m alumina and ultrasonically cleaned for 30 min in absolute ethanol. The NPs were synthesized using a formulation of 85% H_3_PO_4_ and 30% H_2_O_2_ (Sigma-Aldrich, USA) in a 1 : 1 volume ratio at room temperature (RT) for 30 (NP14), 60 (NP7), and 120 min (NP36), to fabricate different diameters. Afterward, the materials were cleaned in ultrapure water for 15 min under sonication, rinsed with ethyl alcohol, and dried before each analysis. The experimental materials were sterilized in a biosecurity cabinet using UV irradiation (285 nm UVB light source) for 30 min each side. Cleaned and sterilized Ti6Al4V foils without any chemical treatment were used as controls for the experimental testing.

### 2.2. Surface Physicochemical Characterization

The surface morphology was analyzed using field-emission scanning electron microscopy (FE-SEM; Tescan LYRA 3) at a 20 kV accelerating voltage with a secondary electron detector. The NP distribution was generated from 50 NPs randomly measured from a FE-SEM micrograph. Energy dispersive X-ray spectroscopy (EDX, Brucker XFlash) coupled to the FE-SEM was used for the chemical analysis. Raman spectroscopy (Raman Station 400F Perkin-Elmer) was applied at RT using a 785 nm diode laser beam at a power of 15 mW. The water contact angle (WCA) was quantified using an automated tensiometer (Theta Attension; Biolin Scientific), placing a 5 *µ*L droplet of deionized water at RT and 45% relative humidity.

### 2.3. Microbial Characterization

We prepared fresh overnight grown cultures of *Staphylococcus aureus* (*S. aureus*, ATCC 25923), *Escherichia coli* (*E. coli*, ATCC 25922), and an isolated *C. albicans* strain as previously described [9]. The active fungal suspension was adjusted to 2 × 10^4^ CFU/mL with Sabouraud dextrose (SD) broth. Then, 50 *µ*L of the working *C. albicans* were cultured over the surfaces, which were individually placed in a 12-well plate (Corning, USA). Similarly, the *S. aureus* and *E. coli* inoculums were tailored to 1 × 10^7^ CFU/mL using tryptic soy (TS) broth and cultured. The materials were incubated for 2 h and 6 h (defined as initial and late adhesion, respectively) at 37°C in static conditions, washed thrice with 1 × phosphate-buffered saline (PBS) for 5 min and ultrasonicated in 2 mL of SD or TS broth [10]. The remaining suspensions were serially diluted and cultured in SD (*C. albicans*) or TS (bacterial cells) agar for 24 h at 37°C.

### 2.4. FE-SEM Microbial Analysis

For FE-SEM analysis, each material was rinsed thrice with warm PBS, fixed in 3% glutaraldehyde (Sigma-Aldrich, USA) at 4°C overnight, rinsed thrice with PBS, and postfixed with 3% glutaraldehyde for 2 h at RT. The samples were dehydrated in a graded series of ethanol for 2 h and placed into a desiccator until the analysis.

### 2.5. Cytotoxicity Assessment Using MTT

In order to analyze the cytotoxicity of the experimental surfaces, we applied the MTT (3-(4,5-dimethylthiazol-2-yl)-2,5-diphenyltetrazolium bromide) viability assay [11]. We used MG-63 human osteoblast-like cells (ATCC CRL-1427). Prior to cell culture, each experimental material was placed in an individual well of a 12-well polystyrene plate (Corning, USA). The initial cell density was 1 × 10^4^ cells/surface in passage three. They were harvested and cultured in complete medium constituted of Dulbecco's modified Eagle's medium (DMEM, Thermo Fisher Scientific, USA) supplemented with 10% heat-inactivated fetal bovine serum (Thermo Fisher Scientific, USA) and 100 units/mL of penicillin-streptomycin (Thermo Fisher Scientific, USA) at 37°C in a humidified 5% CO_2_ incubator for 24 h. Afterward, the cells were washed thrice with warm PBS. 2 mL of MTT (Sigma-Aldrich, USA) in DMEM (5 mg/mL) was added into each well and further incubated at 37°C in a humidified 5% CO_2_ incubator for 3 h. The resulting formazan crystals were dissolved after discarding the medium containing MTT and transferring the 12-well plate into an orbital shaker at 200 rpm, 37°C with dimethyl sulfoxide (Sigma-Aldrich, USA) for 20 min. Then, the dissolved crystals were deposited into a 96-well polystyrene plate (Sigma-Aldrich, USA), and the optical density (O.D.) was recorded at 590 nm using a microplate reader (Thermoskan, Thermo Fisher Scientific, USA).

### 2.6. Statistical Analysis

Numerical data of three independent studies performed each in triplicate were assessed by one-way analysis of variance followed by Tukey's multiple comparison test using GraphPad Prism 7. A *P* < 0.05 was considered statistically significant.

## 3. Results and Discussion

**Figure 1:**
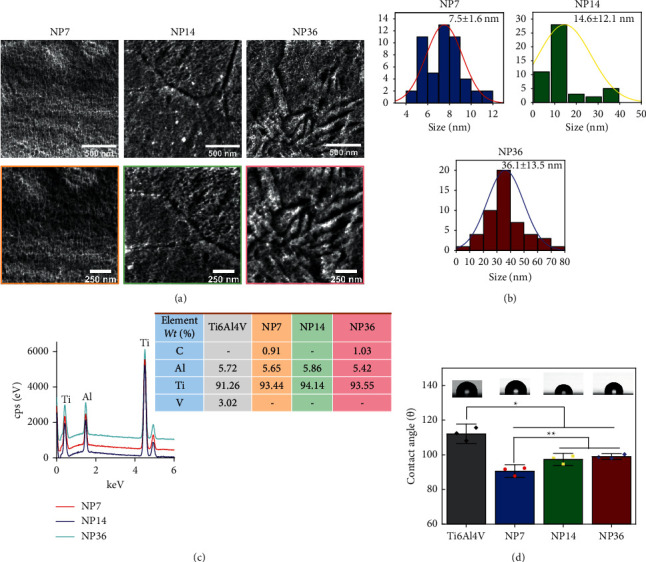
(a) FE-SEM of the synthesized materials. (b) Diameter distribution of the NPs. (c) EDX of the surfaces. (d) WCA of the specimens. The symbols ^*∗*^ and ^*∗∗*^ show significant differences between the materials.

Figure 1(a) illustrates diameter-controlled NPs on Ti6Al4V surfaces after the nanopatterning protocol. Moreover, the high-zoom revealed the formation of homogeneous and ordered nanostructures for each reaction.  Figure 1(b) represents NPs of ≈7 nm (1 h), ≈14 nm (30 min), and ≈36 nm (2 h), indicating that time could be the predominant thermodynamic parameter for NP diameter control at room temperature using the H_3_PO_4_ system. The EDX showed the materials' elemental values (Figure 1(c)), highlighting that phosphorous (P) was not incorporated as a doping complexing element in any treatment. However, EDX is a technology that enables chemical profiling of the X-ray photons generated from the beamed electrons of the deeper layers of the surface [12]. The X-ray photoelectron spectroscopy (XPS) is recommended for clarifying this interesting trend. It is important to note that phosphate coatings could generate electrostatic interactions that might be favorable for promoting bacterial adhesion [13]. Furthermore, the low carbon levels suggest the absence of any organic pollutants (Figure 1(c)). Thus, the lower carbon level and the nanostructured distribution detected on the NPs, especially on NP7, could be attributed to the reduced WCA (Figure 1(d)). The reactions achieved Ti^4+^ by an initial oxidation, followed by the dissolution of the oxide layer, and the nucleation of a thin oxide layer. Thus, decomposing H_2_O_2_ into O_2_ and H^+^ generates nanodefects [14].

(1) $$\begin{array}{c} 2{\text{H}}_{2}{\text{O}}_{2}⟶2{\text{H}}_{2}\text{O}+{\text{O}}_{2}\\ \text{Ti}+2{\text{H}}_{2}{\text{O}}_{2}+4{\text{H}}^{+}⟶{\text{Ti}}^{4+}+4{\text{H}}_{2}\text{O}\\ \text{Ti}+4{\text{H}}^{+}⟶{\text{Ti}}^{4+}+{2\text{H}}_{2}\\ \text{Ti}+{\text{O}}_{2}+2{\text{H}}_{2}\text{O}⟶{\text{Ti}}^{4+}+4{\text{OH}}^{-}. \end{array}$$

**Figure 2:**
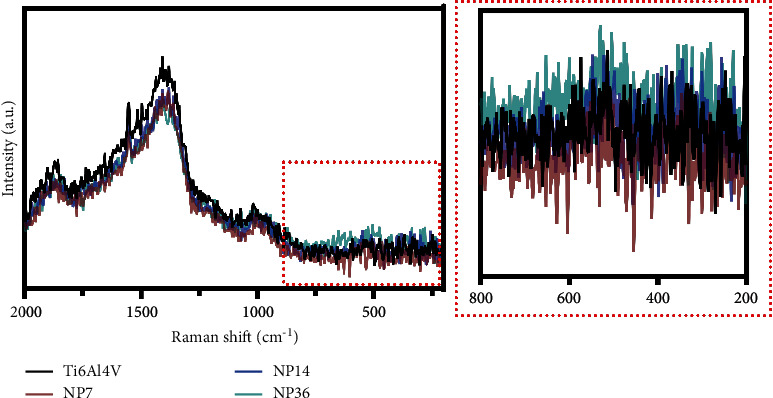
Raman characterization of the experimental materials.

The enlarged atomic vacancies increased the pores, surface area, and free energy of samples. Furthermore, Ti^4+^ generates unstable cationic complexes [Ti_2_O_5_(OH)_2_]^+^, [Ti_2_O_5_(OH)_2_], and [Ti_2_O_5_(OH)_4_]^+^ that decompose into (Ti(OH)_4_) [15]. In a previous study, Pisarek et al. suggested that H_3_PO_4_/H_2_O_2_ at RT after 24 h conducted to form a nanosponge-like surface morphology [16]. Similarly, the authors detected the presence of Ti 2p_3/2_ and O1 s signals that have been ascribed for the Ti-O bond gap, thus proposing the formation of a consistent thick layer mainly of TiO_2_. Interestingly, the Raman analysis (Figure 2) suggests that a thinner TiO_2_ layer could be generated after the nanopatterning process, as there was no bandgap between 800 and 200 nm corresponding to amorphous TiO_2_ [17, 18]. Although the presence of NPs was detected for all the experimental materials, the EDX results also supported this interesting finding, as no oxygen levels were detected. More surface chemistry analyses are recommended in order to support those interesting findings. On the other hand, the study by Pisarek reported that the 24 h treatment phase resulted in the deposition of phosphate ions [16], and far more critical is the fact of possible corrosion detriments by the extensive reaction period. Although this oxidative strategy was applied in previous studies, the role of treatment time for NPs diameter variations was not examined. Previously, Variola et al. synthesized NPs on Ti6Al4V using the piranha solution at RT, interestingly, under extreme caution [1]. The authors showed NP diameters comparable to those of NP7 and NP14. However, the opposite effect was detected in NP36, probably by generating new single nanocavities and pits growing together increasing the diameter. Moreover, it has been described that oxidative etching procedures favor the attack of *ß*-grains over the *α*-grains, therefore altering the surface microtexture over time [1, 19]. Nonetheless, those discrepancies could not be comprehensively harbored in the nanopatterning onsets. Further it is considered that the chemical nature of the acids (H_3_PO_4_ versus H_2_SO_4_) can influence the kinetics and thermodynamics of the reactions. This phenomenon could have been due to the application of a weaker acid such as H_3_PO_4_, which may demand prolonged etching periods as compared to H_2_SO_4_. Correspondingly, this result is in accordance with the thin nanostructured coatings generated on Ti6Al4V using weaker acids/oxidants that also require extreme care protocols [5]. On the contrary, the H_3_PO_4_/H_2_O_2_ system showed hallmarked constant temperature stability beginning from the mixture preparation to each performed reaction. Therefore, we need to take into consideration that H_3_PO_4_ can also work as a stabilizer to suppress the disproportionation of H_2_O_2_ when reacting with trace levels of metal cations [20] and the resulting decomposition [21]. The mixture with H_3_PO_4_ can suppress the H_2_O_2_ reduction resulting in the promotion of H^+^. Furthermore, Shiraishi et al. applying Raman spectroscopy and cyclic voltammetry, suggested that H_3_PO_4_ suppresses the reduction of H_2_O_2_ by a stronger interaction between H_3_PO_4_ and H_2_O_2_ compared with water due to the H-bonding interaction to form an H_2_O_2_-H_2_PO_4_- bidentate complex [22]. The authors also advocated that H_3_PO_4_ associates with H_2_O_2_ via H-bonding to form a stabilized complex, which may inhibit the H_2_O_2_ reduction and decrease the enthalpy required to conduct an exothermic reaction, as observed here, thus far proposing that H_3_PO_4_ is safer and more manageable than H_2_SO_4_.

**Figure 3:**
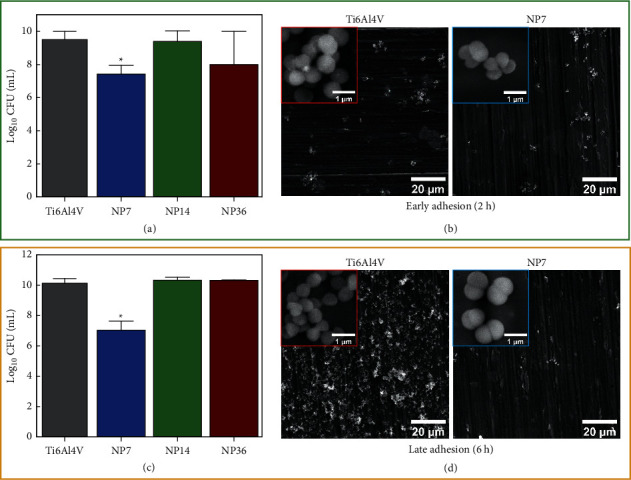
Antibacterial behavior on the experimental materials. (a) Bacterial viability after 2 h of incubation. (b) *S. aureus* morphology on the control and NP7 material at early adhesion phase. (c) *S. aureus* growing viability after 6 h. (d) *S. aureus* phenotype characterization at the late adhesion. The symbol ^*∗*^ shows significant cell viable differences between the material groups.

**Figure 4:**
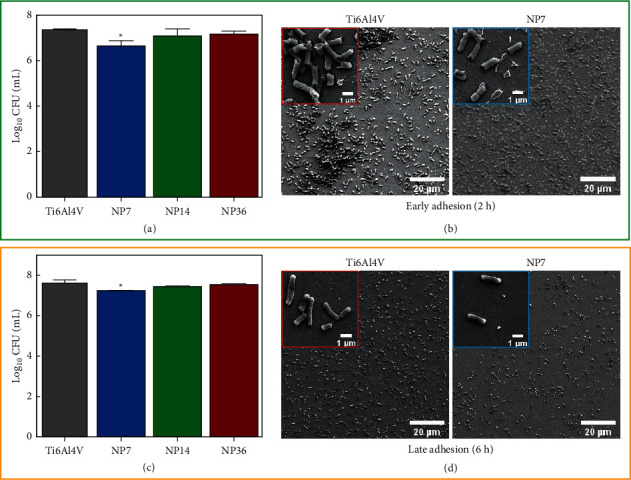
Antibacterial behavior of *E. coli* on the experimental materials. (a) Viability evaluation after 2 h of incubation. (b) *E. coli* morphology on the control and NP7 material at early adhesion phase. (c) *E. coli* growing viability after 6 h. (d) *E. coli* phenotype characterization at the late adhesion. The symbol ^*∗*^ shows significant cell viable differences between the material groups.

**Figure 5:**
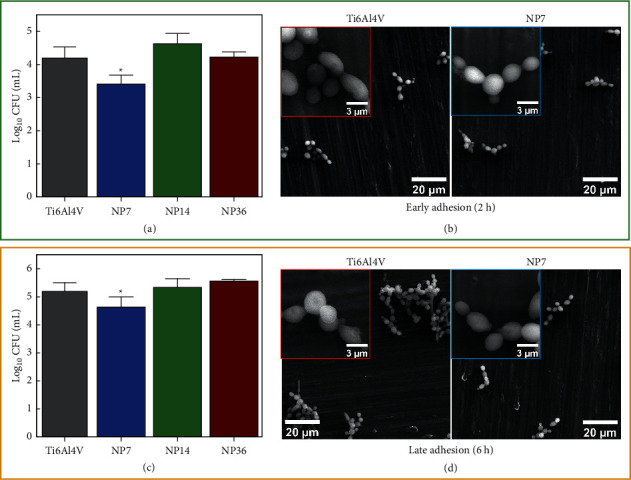
Antifungal analysis on the materials specimens. (a) *C. albicans* viability behavior after 2 h of growth. (b) Fungal morphology characterization on the control and NP7 surfaces at early adhesion phase. (c) *C. albicans* viability counts after 6 h of incubation. (d) Fungal phenotype evaluation at the late adhesion stage. The symbol ^*∗*^ illustrates significant differences among the experimental groups.

Medical implant contamination is a paramount concern that negatively compromises the biomaterials' “gold success”: achieving complete clinical healing and restoration [23], thus highlighting that nanotextured surfaces are an important and acceptable strategy to reduce microbial adhesion. Our results suggested that smaller NPs (NP7) could avoid the *S. aureus* adhesion for each growing phase (Figure 3). Interestingly, similar outcomes of bacterial colonization were detected for the NP14, NP36, and control. Moreover, we can focalize that SEM micrographs illustrated an analogous spherical phenotype commonly observed for coccus bacteria (Figure 3, insets). Furthermore, the micrographs suggest that higher biofilm colonization agrees with the increased viability detected. A similar behavior was detected for the *E. coli* model evaluated on the experimental materials (Figure 4). The *E. coli* growing ability was reduced on the NP7 material in comparison with the study surfaces. Of particular interest is the fact of similar bacterial morphology conducted by the Ti6Al4V alloy and the NP7. The high-zoom micrographs (Figure 4, insets) clearly show that bacilli configuration is present in plenty on the evaluated surfaces. However, from the low-zoom micrographs, we can highlight that the NP7 reduced the bacterial adhesion, as mainly detected at the late adhesion phase. Importantly, the NP7 supported antifungal behavior (Figure 5), following comparable results to those of *S. aureus* and *E. coli*. In the early adhesion, we detected that the control caused enlarged cell alterations, which were further transformed into hyphae and pseudohyphae morphologies. Meanwhile, NP7 conserved a downregulated proliferating phenotype. Principally, we discovered a substantially growing fungal viability for the larger NPs, further proposing that smaller NPs can present detrimental fungal outcomes, as previously reported [24]. Notably, these results suggest that the smaller NPs could disrupt the formation of nanoscale bonds required to conduct a proper microbial adhesion, in accordance with previous works [9, 25]. In particular, the significant hydrophilicity on NP7 may positively influence the reduced electrostatic interactions for microbial bonding [26] and the amorphous nature of the TiO_2_ thin coating [27]. However, we recommend more physicochemical studies to explain the current antimicrobial results.

**Figure 6:**
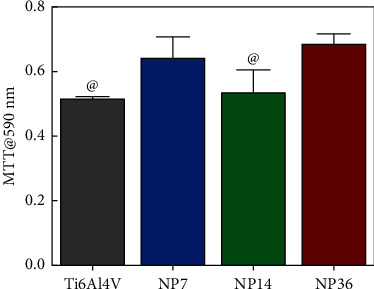
Osteoblast viability assessment after 24 h on the experimental materials. The symbol @ indicates statistical differences against NP7 and NP36.

It is well known that the modification of a conventional surface material to its nanostructured counterpart can alter the cellular activity [2, 28, 29]; a critical result presented from this work is the finding that nanoporous surfaces developed by etching with an H_3_PO_4_/H_2_O_2_ mixture can improve osteoblast activity. In Figure 6, it is presented the osteoblast viability on the experimental materials after 24 h of culture. The results suggested that the NP7 and NP36 nanostructured surfaces promoted higher cellular proliferation in contrast to the control alloy and the NP14. It is essential to consider that MTT assays take advantage of mitochondrial activity, pointing toward the fact that a higher quantification of resulting formazan crystals is directly proportional to a healthy and active osteoblast growing population [30, 31]. Therefore, we can hypothesize that nanoporous distribution may play a more critical role in promoting early osteoblast proliferation instead of the NP size. This information can be in part supported by the fact that NP7 and NP36 share a more distributed porous size than NP14. On the other hand, NP7 showed higher osteoblast activity and more hydrophilicity, indicating improved surface energy and enhanced early cellular growth. This current trend opens the concept that a high surface-area-to-volume ratio and improved surface energy can establish a stimulating microenvironment that can accelerate the bone-growing functionality, as depicted in previous studies of different size-controlled nanostructured coatings [32–35].

## 4. Conclusions

Our results established an oxidative nanopatterning protocol using H_3_PO_4_/H_2_O_2_ as a feasible, safer, and controllable system for developing homogeneous nanotextured surfaces on Ti6Al4V. The H_3_PO_4_/H_2_O_2_ resulted in a stable mixture that did not show violent exothermic reactions during the preparation of the solutions and the alloy surface modification. Inherently, our protocol resulted in NPs of 7, 14, and 36 nm outlining the reaction time as the main variable for diameter control under the studied synthetic conditions. Importantly, we have demonstrated that smaller NPs can reduce the early adhesion of *S. aureus*, *E. coli*, and more strikingly, *C. albicans*. Thus, more attractively, NP7 tailored a long-lasting antimicrobial action for 6 h of incubation, particularly with the absence of cellular phenotype alterations. On the other hand, the cytotoxicity analysis suggested that the surfaces might not disrupt the initial osteoblasts' proliferation, tailoring the nanostructures as a stable surface for osteoactive conditions. Our work opens a new path for the rationale design of nanobiomaterials with “intelligent surfaces” capable of decreasing microbial adhesion and being scalable for industrial transfer.

## Data Availability

All data generated or analyzed in this study are included in this work.
